# Identifying chronic pain subgroups in the UK biobank for persona development: A clustering analysis

**DOI:** 10.1177/20552076251333497

**Published:** 2025-05-14

**Authors:** Ting-Chen Chloe Hsu, Pauline Whelan, Christopher J Armitage, John McBeth

**Affiliations:** 1 Centre for Musculoskeletal Research, 5292 University of Manchester, Manchester, UK; 2 Centre for Health Informatics, Division of Informatics, Imaging & Data Sciences, 5292 University of Manchester, Manchester, UK; 3 Manchester Centre for Health Psychology, 5292 University of Manchester, Manchester, UK; 4 NIHR Greater Manchester Patient Safety Research Collaboration, 5292 University of Manchester, Manchester, UK; 5 The NIHR Manchester Musculoskeletal Biomedical Research Unit, 5293 Central Manchester University Hospitals NHS Foundation Trust, Manchester, UK; 6 School of Primary Care, Population Sciences and Medical Education, 7423 University of Southampton, Southampton, UK

**Keywords:** Chronic pain, clustering analysis, persona, UK Biobank, digital health interventions

## Abstract

**Purpose:**

To conduct a preliminary clustering analysis using the UK Biobank to (1) identify distinct chronic pain clusters based on age, sex, and number of pain sites; (2) assess the associations between chronic pain clusters and health-related outcomes; and (3) outline future directions for developing chronic pain personas to inform targeted digital health interventions.

**Methods:**

Participants were selected from a 2019 chronic pain survey. The domains included demographics, pain, daily functioning, and emotional health. The clustering analysis employed the k-prototype algorithm. Cluster characteristics were summarised and quantified using multinomial logistic regression. Preliminary data personas were described.

**Results:**

89,853 people with chronic pain were analysed (60.4% female, mean age 66.5 years). Five clusters were identified: Fibromyalgia-like pain (FP, 11.2%), multisite pain (MP, 17.9%), younger with regional pain (21.9%), middle age with regional pain (MRP, 25.5%), and elderly with regional pain (ERP, 23.5%). FP was associated with more severe health-related outcomes, characterised by greater depression, fatigue, and difficulties with daily activities and social relationships. Sleep, mobility, and usual activities were commonly affected at mild and moderate levels across all clusters. Fatigue and depression varied, with FP and MP experiencing greater impacts. ERP and MRP were associated with a lower likelihood of adverse health-related outcomes.

**Conclusion:**

All chronic pain clusters identified from the UK Biobank showed common challenges in sleep, mobility and daily functioning; the impacts of fatigue and depression varied between clusters. The next step involves engaging key stakeholders to create, refine, and validate these personas to inform the development of targeted digital health interventions.

## Introduction

Digital health interventions (DHIs) have gained increasing popularity for their potential to transform healthcare delivery. By leveraging digital technologies such as computers, smartphones, and wearables, DHIs improve access to care by reducing geographical and time barriers, and enhance the efficiency of care delivery through real-time data management.^1^ Particularly beneficial in chronic disease management, DHIs are used to support care continuity and self-management at lower costs.^2^ However, developing effective DHIs can be challenging due to their complex and multicomponent nature.^3^ These interventions often serve multiple purposes (e.g., engagement, risk assessment, behaviour modification) and incorporate various behaviour change techniques (e.g., goal setting, self-monitoring, social comparison). A well-designed DHI needs to achieve a balance between functionality and user experience, underpinned by rigorous scientific evaluations of its efficacy and effectiveness.^4^

Involving key stakeholders in the development of DHIs is essential to maximise their positive impact.^5^ This process, known as co-design, is a collaborative approach whereby stakeholders such as patients, clinicians, academics, and policy makers are actively involved in the development, implementation and/or evaluation.^6^ Co-design enables researchers to identify user needs, address barriers, and tackle inequalities. However, co-design is an iterative process, and managing its lifecycle requires substantial time and resources. Challenges may include obtaining ethical approvals, recruiting suitable participants, managing the logistics of co-design activities, and maintaining participant engagement. Previous research has reported that establishing shared values, understanding and expectations with stakeholders is critical, as is building an equal knowledge base and maintaining flexible decision-making power.^7^ Co-design processes need to leverage agile approaches to enhance efficiency, scalability, and representativeness, while also facilitating rapid updates and modifications.

Personas, a co-design method, are exemplary user profiles that are increasingly adopted in product design and research.^8^ They serve as practical tools for creating memorable, interactive, engaging, and actionable representations of prospective users to guide the design process. Persona development can be guided by the persona lifecycle framework,^9^ which comprises five phases: family planning (identify the purpose and data sources), conception and gestation (define assumptions and create personas), birth and maturation (introduce personas to the team), adulthood (use personas in the design, development, evaluation, and release of DHIs), and lifetime achievement and retirement (assess the effectiveness of personas and plan their future reuse or retirement). There are three types of personas: proto-personas, user personas, and data personas.^10^ Proto-personas are preliminary assumptions based on indirect interactions with users, providing a starting point for further research and development. User and data personas, derived from direct user research, offer a more detailed understanding of user characteristics, behaviours, attitudes, motivations, needs, and goals. While personas often include fictional narratives to appear more human-like, their archetype structures and attributes (e.g., demographics, behaviour patterns) are typically based on data collected from target users. Personas have been used in the development of DHIs for chronic disease management, such as stress reduction for cancer,^11^ dietary management for obesity,^12^ and treatment adherence for cardiovascular diseases.^13^ The methodologies for creating personas vary and may employ either a single data source or an iterative approach using multiple sources to enrich the profiles. Common data collection techniques include surveys, focus groups, interviews, ethnographic observations,^14^ or the use of existing databases such as electronic health records.^15^ Data analysis may involve approaches such as thematic analysis^12^ or clustering analysis.^16^ The method chosen for persona development depends on design purposes and target users, but the ultimate goal is to define distinct and informative user groups that can effectively inform the development of DHIs.

In chronic pain, personas have not been widely used in developing DHIs. A scoping review of ten DHIs that employed personas reported that while most were transparent about their methodologies, only half provided details of their developmental processes.^17^ Research has demonstrated that DHIs are effective in short term for reducing pain severity and improving pain impacts such as physical functioning, emotional well-being, and sleep quality.^18–20^ However, chronic pain is a highly heterogeneous and variable condition. For instance, pain variability within and across individuals leads to different pain trajectories, which are associated with varying levels of disability, psychological distress, and work absence.^21^ Individual characteristics such as age, sex, and pain sites are also associated with varied outcomes in pain severity, disability, and psychological distress.^22,23^ Pain researchers have suggested that interventions customised for subgroups with similar characteristics may produce better outcomes,^24,25^ as these subgroups exhibit unique treatment needs and respond differently to treatments.^26,27^ Personas may be a promising approach to represent the heterogeneity of chronic pain subgroups and guide the development of DHIs that can provide support targeted at subgroup levels.

To begin applying personas to the development of targeted DHIs for chronic pain, this study aimed to conduct a clustering analysis using the UK Biobank database as the first step in identifying distinct patterns in chronic pain. It is situated in the persona conception and gestation phase, during which a suitable data source was identified and used to generate preliminary data personas that capture high-level patterns of chronic pain in the UK Biobank. The objectives included (1) identifying distinct clusters of chronic pain based on age, sex, and number of pain sites; (2) assessing the associations between chronic pain clusters and various health-related outcomes, including depression, fatigue, sleep quality, cognitive functions, and mobility; and (3) providing recommendations for the next steps in creating chronic pain personas to guide the development of targeted DHIs.

## Methods

### Study population

The UK Biobank is a large-scale, population-based prospective study that provides extensive, de-identified health-related information from over half a million UK participants (*N* = 502,283), primarily of middle age and older.^28^ The database contains multiple domains such as genetics, neuroimaging, physical and mental health, medical history, environmental factors, and lifestyle, with ongoing follow-up for a wide range of health-related outcomes. Baseline assessments from 2006 to 2010 involved individuals aged 40–69 from Scotland, England, and Wales. Subsequent repeated assessments and additional evaluations on cohort subsets included multimodal imaging and multi-dimensional surveys on topics such as diet and digestive health, cognitive functions, occupational history, mental health and wellbeing, chronic pain, sleep, and social interactions. Up to May 2017, there were 1297 participants lost to follow-up. The reasons include leaving the UK, withdrawing consent for future linkage, and death.

### Data selection

This study utilised baseline assessments and a 2019 online follow-up survey on chronic pain in the UK Biobank (data dispensed in November 2023). Baseline information included sex, date of birth, ethnicity, age completed full-time education, and the index of multiple deprivation (IMD) for Scotland, England and Wales.^29^ The pain questionnaires^30^ included medical conditions, pain locations, health-related quality of life, depression, and fatigue. Exclusions from the pain questionnaires were nature of pain, specific sections for headache, legs and feet, and pain impact. These sections were either not directly relevant to this study or only recorded answers from subsets of participants who met the criteria for those sections. Table 1 shows the domains and subdomains, along with Field IDs that indicate the questions used in the analysis.

**Table 1.** Domains and subdomains used in the analysis.

Domains	Subdomains	Field IDs in the UK Biobank
Demographic	Sex	31
	Age	34, 54, 120,128
	Ethnicity	21,000
	Age completed full-time education	845
	Index of multiple deprivation	26,140, 26,426, 26,427
Pain	Duration of pain	120,020
	Number of pain sites	21, 23–31, 33–36
	Predominant pain site	120,037
	Number of pain-associated conditions	120,000, 120,001, 120,003– 120,005, 120,007– 120,011, 120,016– 120,018
Depression	Had depression in the past 6 months	120,044
	PHQ-9	120,104– 120,112
	Difficulty of depression	120,113
Cognition	Cognitive symptoms (problems with memory, thinking skills and/or concentration)	120,042
Sleep	Waking unrefreshed	120,041
Fatigue	Have fatigue for at least 6 months	120,114
	Interference with physical functioning	120,122
	Among three most disabling symptoms	120,126
	Interference with work, family or social life	120,127
Health outcomes	Mobility	120,098
	Self-care (washing or dressing)	120,099
	Usual activities (work, study, housework, family or leisure activities)	120,100
	Overall health	120,103

### Demographic information

Demographic variables comprised sex (male, female), age, ethnicity (White, Mixed, Asian or Asian British, Black or Black British, Chinese, other ethnic group), age completed full-time education, and IMD score decile for Scotland, England and Wales (from the least to most deprived). Age was calculated using the date of birth (month, year) and the date when the chronic pain survey was completed. IMD scores were converted to deciles scale using R due to variations in metrics across countries. The scores for three countries (England, Scotland, Wales) were calculated independently by employing the ntile function from the dplyr package which evenly distributed the scores into ten groups for each country. These scores reflected a combination of domains including income, employment, health, education, crime, service accessibility, housing, and living environment.

### Pain information and associated impact

Pain-related variables included duration of pain (3–12 months, 1–5 years, and more than 5 years), number of pain sites (score range 0–14), predominant pain sites, number of health conditions (score range 0–13), depression, cognitive symptoms, sleep refreshment, fatigue, and health outcomes.

The assessment of pain sites consisted of pain all over the body, head, face, neck or shoulders, back, stomach or abdomen, hips, knees, arms, hands, feet, legs, chest, and other body parts. Participants indicated the presence of pain in each location. The number of pain sites was summarised on a scale from 0 (no pain in any of the specified locations) to 14 (pain all over the body). Participants who reported having pain all over the body were not asked subsequent questions about other specific pain locations.

Health conditions associated with chronic pain included osteoarthritis, rheumatoid arthritis, carpal tunnel syndrome, complex regional pain syndrome, chronic post-surgical pain, diabetes, nerve damage/neuropathy other than diabetic neuropathy, fibromyalgia, chronic fatigue syndrome, gout, migraine, pelvic pain, and post herpetic neuralgia. Participants indicated the presence of each condition, which was then summarised.

Depression was assessed using the patient health questionnaire (PHQ-9, score range 0–27, score ≥ 10 indicates major depression),^31^ along with questions about the presence of depression symptoms in the past six months (yes, no) and the difficulty of these symptoms over the past two weeks (not at all, somewhat, very, extremely). Depression was also categorised based on PHQ-9 scores into none (0–4), mild (5–9), moderate (10–14), moderately severe (15–19), and severe (20–27).

Cognitive symptoms and sleep refreshment were evaluated by their perceived severity of the problem (no, mild, moderate, severe). Fatigue-related questions^32^ assessed the presence of persistent or recurrent tiredness, weariness or fatigue lasting for at least six months (yes, no). Participants also indicated their level of agreement on a 7-point scale (from disagree to agree) regarding the extent to which fatigue interferes with physical functioning, work, family, or social life, and whether fatigue ranks among their top three most disabling symptoms.

Health outcomes were measured using EQ-5D-5L,^33,34^ which assessed the perceived severity of problems on mobility, self-care, and usual activities (no, slight, moderate, severe, unable). Participant also rated their overall health on the day using a vertical visual analogue scale (0–100).

### Participant inclusion and exclusion criteria

Participants were selected based on their responses to two questions: whether they have had cancer pain (Field ID 120002 in the UK Biobank) and whether they are troubled by pain or discomfort, either all the time or on and off, that has been present for more than three months (Field ID 120019). The possible responses to these questions were “yes”, “no”, “do not know”, or “prefer not to answer”. To be included in the analysis, participants were required to (1) never have had cancer pain (answer “no” to Field ID 120002) and (2) have experienced pain or discomfort for more than three months (answer “yes” to Field ID 120019). Participants were excluded if they responded “yes” to having had cancer pain, “no” to having experienced pain or discomfort for more than three months or provided “do not know” or “prefer not to answer” to either question.

### Statistical analysis

All analyses were conducted using the UK Biobank research analysis platform. The analysis involved two steps. First, the k-prototypes algorithm,^35,36^ capable of handling mixed numeric and categorical data, was used in Python to perform clustering analysis and identify subgroups based on sex, age, and number of pain sites. Both age and the number of pain sites were standardized. The k-prototypes algorithm calculated dissimilarity by measuring the differences between the data points within each cluster and their respective cluster centroids. This quantified the homogeneity within each cluster. The elbow method was then used to visually determine the optimal number of clusters, identified at the point where increasing the number of clusters yields diminishing returns in reducing the dissimilarity cost. Next, we used R to conduct descriptive analysis to characterise the clusters, followed by multinomial logistic regression^37^ to assess the associations of clusters and health-related outcomes. Results were presented as relative risk ratio and 95% confidence interval (CI).

### Ethical approval

This study was supported by the Chronic High Impact Pain and UK Biobank study (UK Biobank application: 98481, grant reference: MR/W026872/1), which aims to identify possible causes or risk factors for high-impact chronic pain. The UK Biobank study was approved by the National Research Ethics Service (11/NW/0382).

## Results

Of the 502,283 UK Biobank participants, 89,853 with chronic pain were analysed. Among these, 60.4% were female, 96.7% identified as white ethnicity, and the mean age was 66.5 years (SD = 7.6, range = 48.8–83.8). The mean age completed full-time education was 17.1 years. The IMD median scores of England, Scotland and Wales were 11.7 (IQR 6.9–20.7), 6.8 (IQR 3.8–15), and 9 (IQR 5.6–17.4). An equal proportion reported living with chronic pain for 1–5 years (37.5%) and more than 5 years (37.7%). The most bothersome pain sites for participants were back (14.1%), knees (12.6%), and neck or shoulders (11%), whereas 10.1% reported having pain all over the body. The most frequent pain-associated conditions were osteoarthritis (30.2%), followed by migraines (16.1%) and non-diabetic neuropathy or nerve damage (9.9%).

### Optimal number of clusters

The results of the k-prototypes algorithm showed that the elbow point occurred at *k* = 5, suggesting that five clusters optimally minimised the total dissimilarity cost, with further increases in the number of clusters providing diminishing improvements (Additional file 1). Five-clusters is an appropriate choice for this cohort, reducing the total dissimilarity cost from 131,738.3 to 53,833.8.

### Cluster characteristics

The five clusters were categorised into widespread pain and regional pain. The widespread pain category comprised two clusters. Fibromyalgia-like pain (FP, 11.2%) comprised the majority of individuals (90.4%) who reported having pain all over the body across all ages. Multisite pain (MP, 17.9%) included individuals aged over 60 years who all reported pain at multiple sites. The regional pain category included three clusters. Younger with regional pain (YRP, 21.9%) encompassed a range from single-site to MP with younger individuals under 65 years. Middle age with regional pain (MRP, 25.5%) consisted of individuals between 60 and 70 years with pain at fewer sites. Elderly with regional pain (ERP, 23.5%) included predominantly male individuals (67.1%) with fewer pain sites, all of whom were over 65 years. Full cluster characteristics are provided in additional file 2.

### Demographics

**Figure 1.**
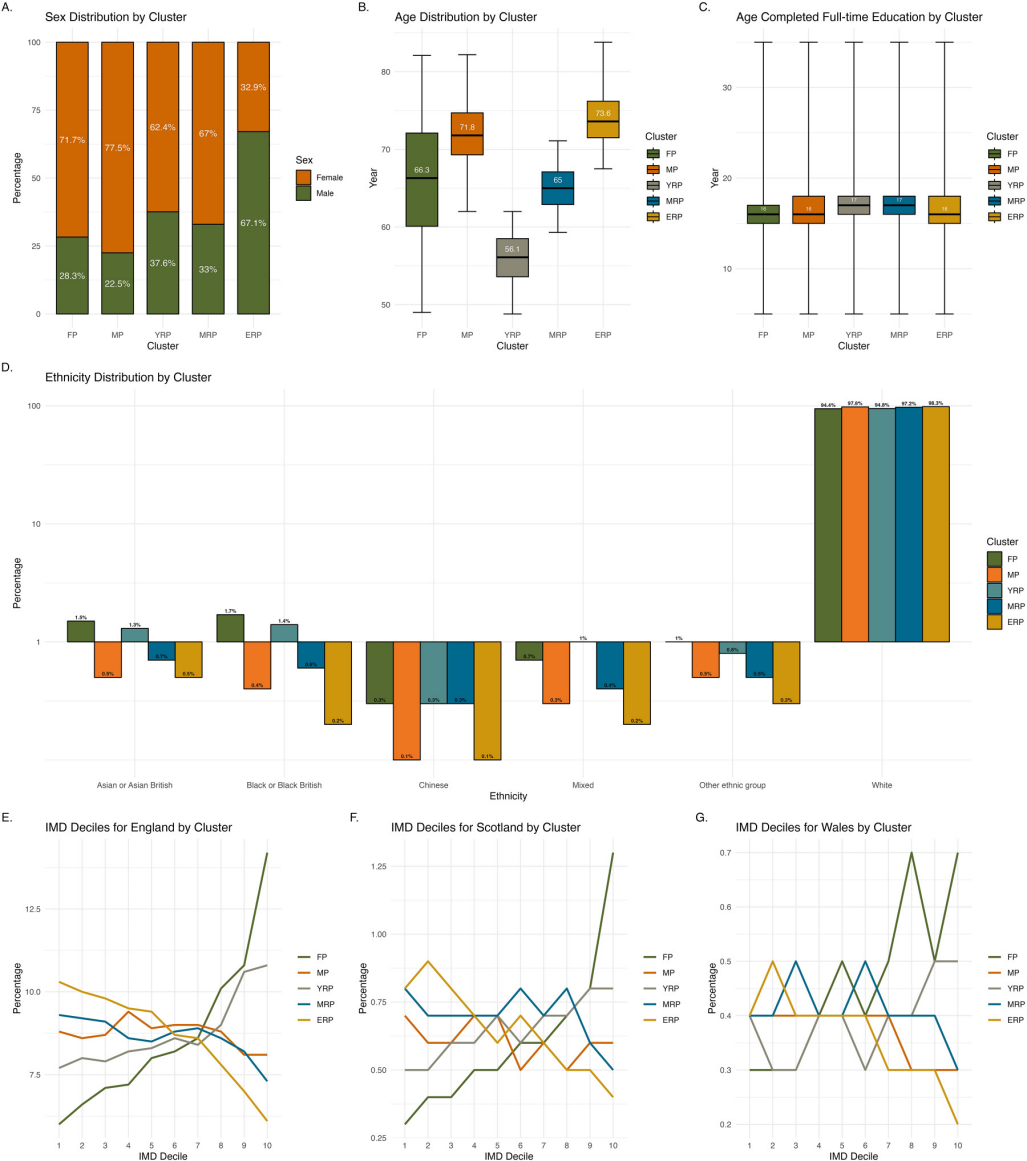
Demographic characteristics across clusters. Illustrates the demographic characteristics across clusters, with different panels representing specific attributes: sex (Panel A), age (Panel B), age completed full-time education (Panel C), ethnicity (Panel D), and index of multiple deprivation by country (Panels E, F, G). The *y*-axis in Panel D is scaled proportionally where values below 1% are represented in expanded format to enhance visibility of smaller percentages (e.g., 0.1% appears more extended from 1% to emphasise lower frequency, while higher values like 0.7% are comparatively closer to 1%).

Figure 1 shows the demographic characteristics across clusters. Females predominated in FP (71.7%) and MP (77.5%), whereas males predominated in ERP (67.1%). Both FP and ERP were characterised by older individuals, with a median age of over 70 years, while YRP mainly included slightly younger individuals in their 50 s. FP and YRP had the highest proportion of participants from non-white ethnicities (around 5% each), primarily identifying as Black or Black British and Asian or Asian British. The median age of completing full-time education is similar across all clusters, at 16–17 years. FP and YRP showed greater deprivation, with more than one-third of each cluster classified in the higher IMD deciles. In contrast, ERP showed the least deprivation among the clusters, with over one-third in the opposite IMD deciles.

### Pain

**Figure 2.**
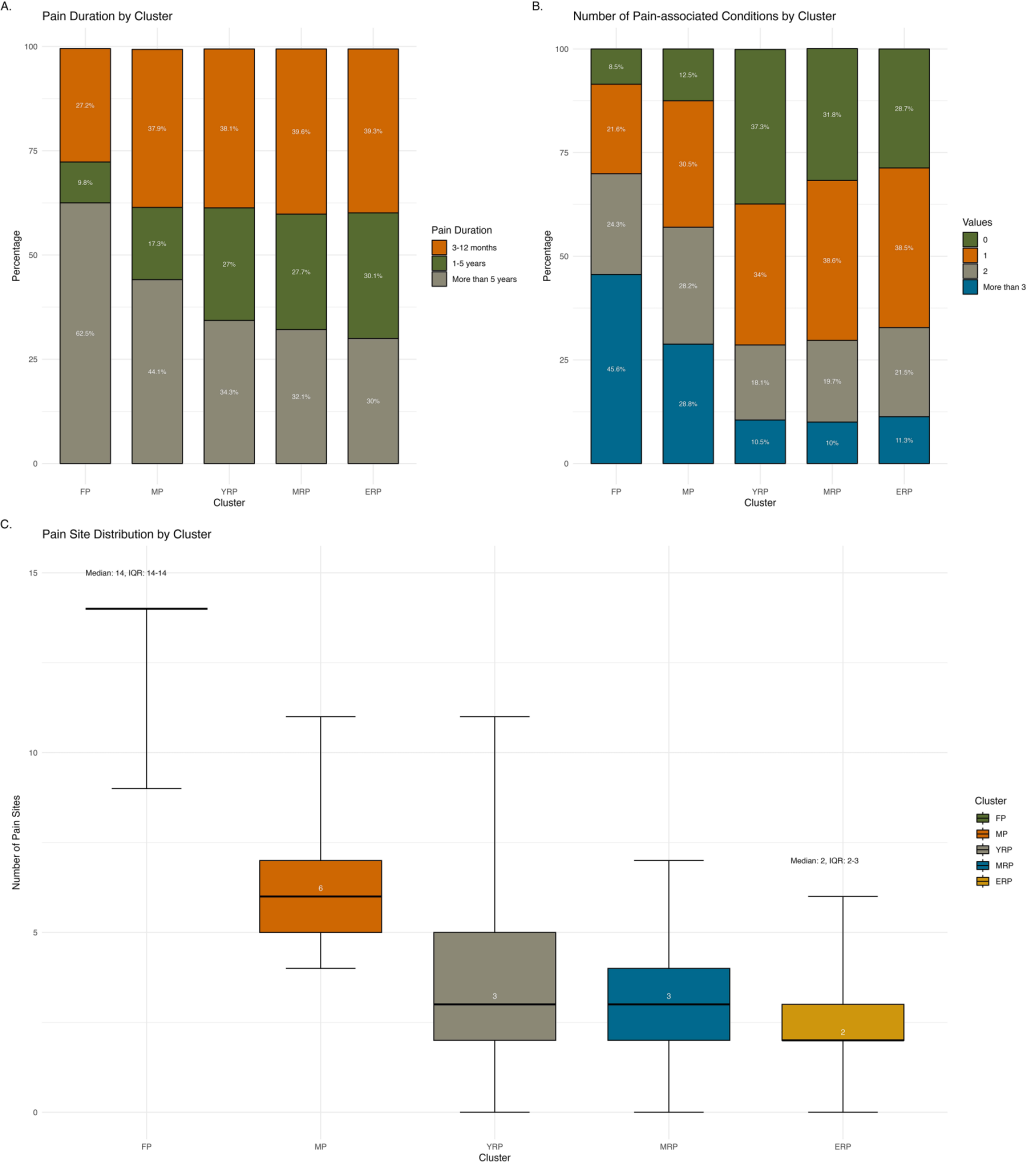
Pain characteristics across clusters. illustrates pain characteristics across clusters, including duration of pain (Panel A), number of pain-associated conditions (Panel B), and number of pain sites (Panel C).

Figure 2 shows the pain characteristics across clusters. All individuals in FP and MP reported MP, ranging from four sites to pain all over the body. In contrast, individuals in the remaining clusters reported fewer pain sites, with a median of two or three sites. Apart from FP, individuals in other clusters were mostly bothered by pain in the back, knees, and neck or shoulders. More than half of the individuals in FP (62.5%) have lived with pain for more than five years, whereas 44.1% in MP reported the same duration. The duration of pain in the remaining clusters were more evenly distributed, but over one-third with pain lasting between one to five years. Nearly half of the individuals in FP (45.6%) reported having three or more pain-associated conditions. MP showed an even distribution, with about one-third experiencing either one, two, and three or more conditions each. In the remaining clusters, the majority of individuals (over 60%) had only one or no pain-associated conditions. The most common pain-associated conditions were osteoarthritis, migraine, and nerve damage or non-diabetic neuropathy across all clusters. In FP and MP, over 60% reported having osteoarthritis, and about one-third reported experiencing migraines. We also observed a higher proportion of rheumatoid arthritis and gout in MRP and ERP.

### Health-related outcomes

**Figure 3.**
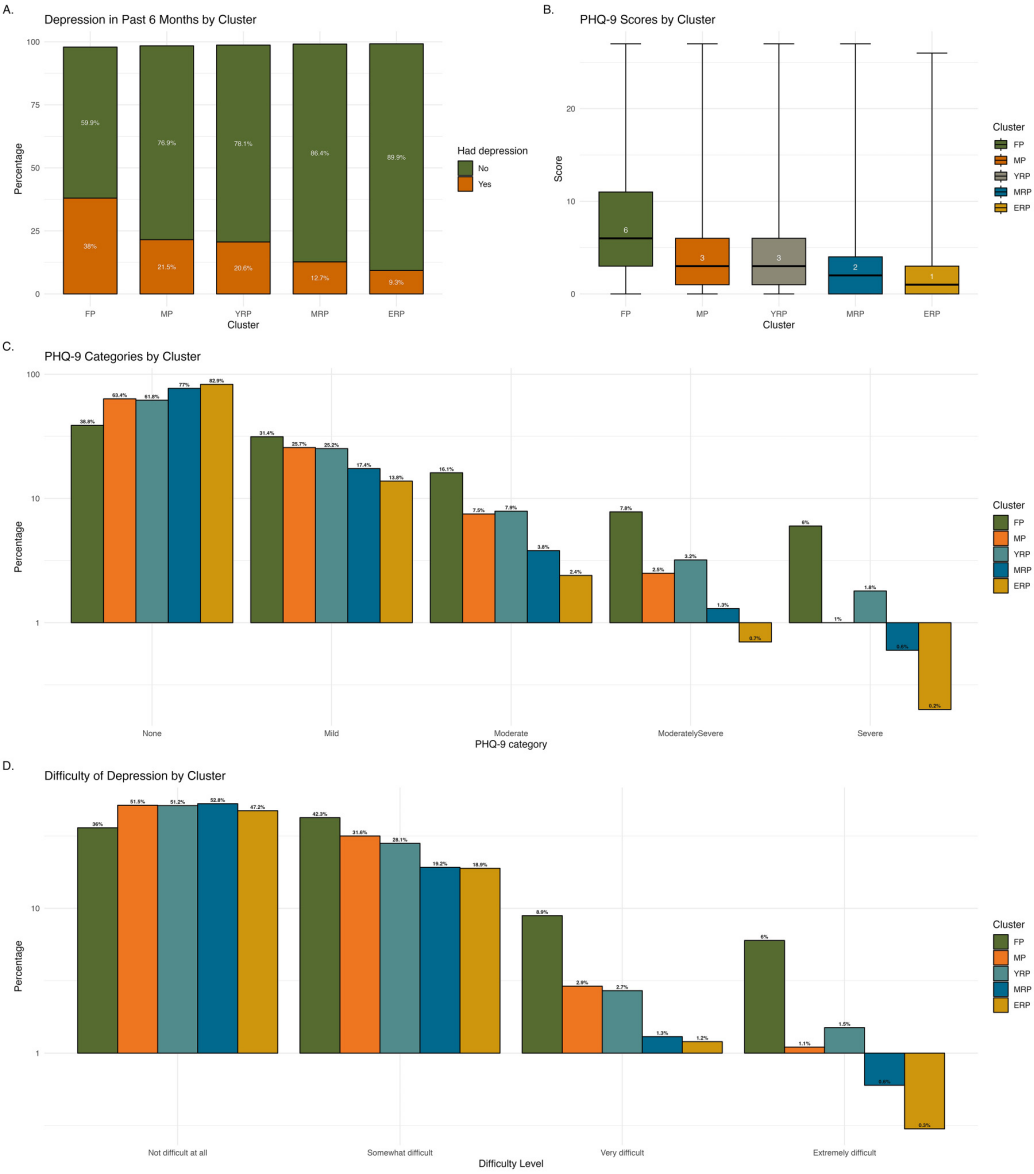
Characteristics of depression across clusters. Illustrates the characteristics of depression across clusters, including had depression is the past 6 months (Panel A), patient health questionnaire (PHQ)-9 scores (Panel B), PHQ-9 categories (Panel C), and difficulty of depression (Panel D). The *y*-axes in Panels C and D are scaled proportionally where values below 1% are represented in expanded format to enhance visibility of smaller percentages. (e.g., 0.1% appears more extended from 1% to emphasise lower frequency, while higher values like 0.7% are comparatively closer to 1%).

Depression (Figure 3) over the past six months was common in FP (38%), but much less in ERP (under 10%). Individuals in FP mostly experienced mild to moderate depression (47.5%; PHQ-9 median score: 6, IQR: 3–11), with 13.8% falling into moderately severe and severe categories. Approximately one-third of individuals in MP and YRP reported mild to moderate depression, while less than 20% did so in MRP and ERP. Their median PHQ-9 scores indicated few signs of depression. Over half of FP (57.2%) reported that depression caused difficulties with work, home responsibilities, or social interactions, with 14.9% finding it very to extremely difficult. In comparison, around one-third in MP and YRP reported having difficulties, which decreased to about 20% in MRP and ERP.

**Figure 4.**
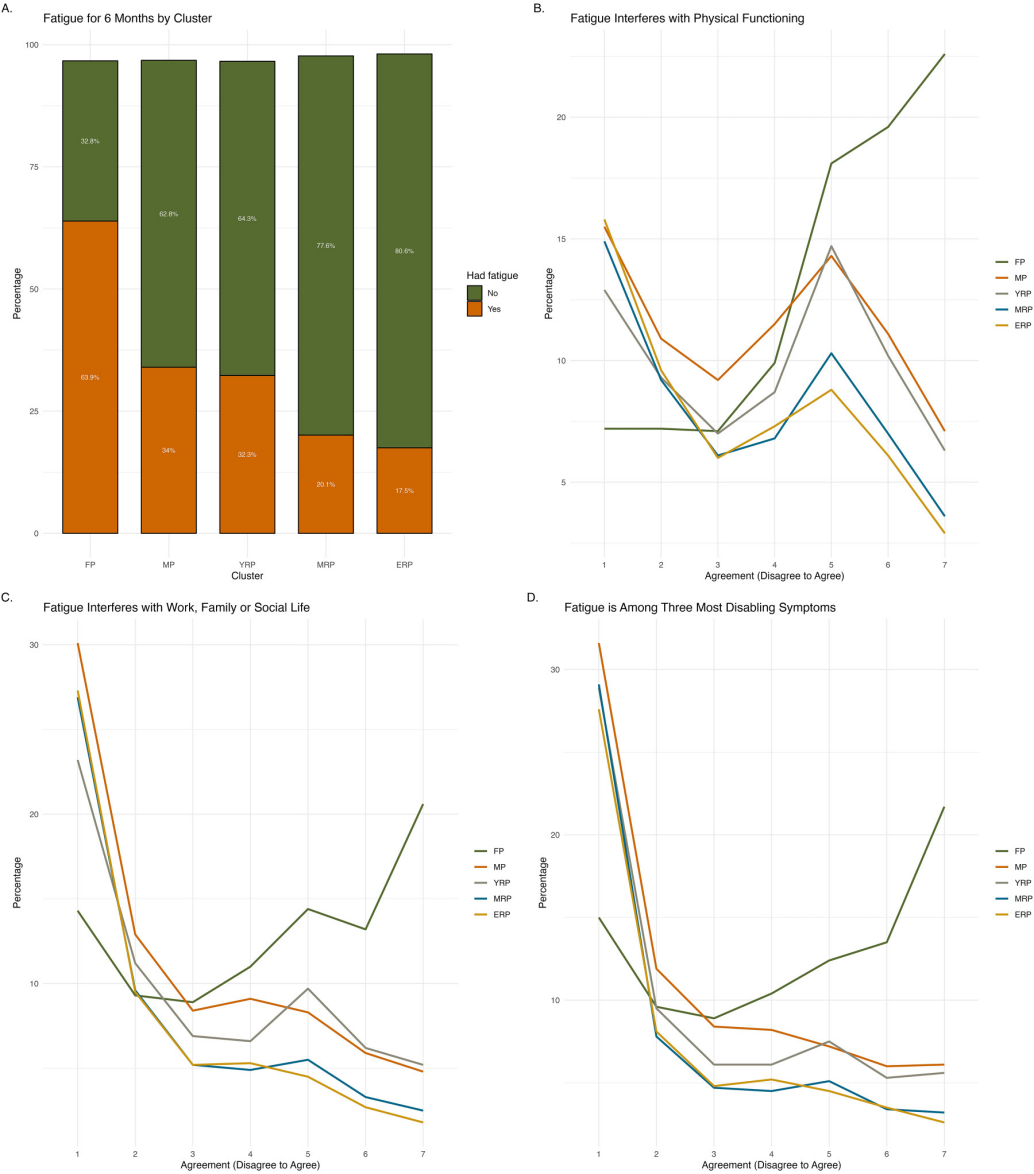
Characteristics of fatigue across clusters. Illustrates the characteristics of fatigue across clusters, including had fatigue for more than 6 months (Panel A), fatigue interferes with physical functioning (Panel B), work, family or social life (Panel C), and fatigue is among the top three most disabling symptoms (Panel D).

Fatigue (Figure 4) lasting for six months was more prevalent in FP (63.9%), decreasing to one-third in MP and YRP, and about 20% in the remaining clusters. Among those experiencing recent fatigue, the majority in FP agreed (scored above 5 on a 7-point scale) that it interfered with their physical functioning (60.3%) and their work, family, or social life (48.2%). Another 47.6% identified fatigue as one of their top three most disabling symptoms. MRP and ERP reported the lowest proportions (about 10%) regarding fatigue impact and its importance as a top symptom, while MP and YRP showed roughly 20%–30% agreement on these aspects.

**Figure 5.**
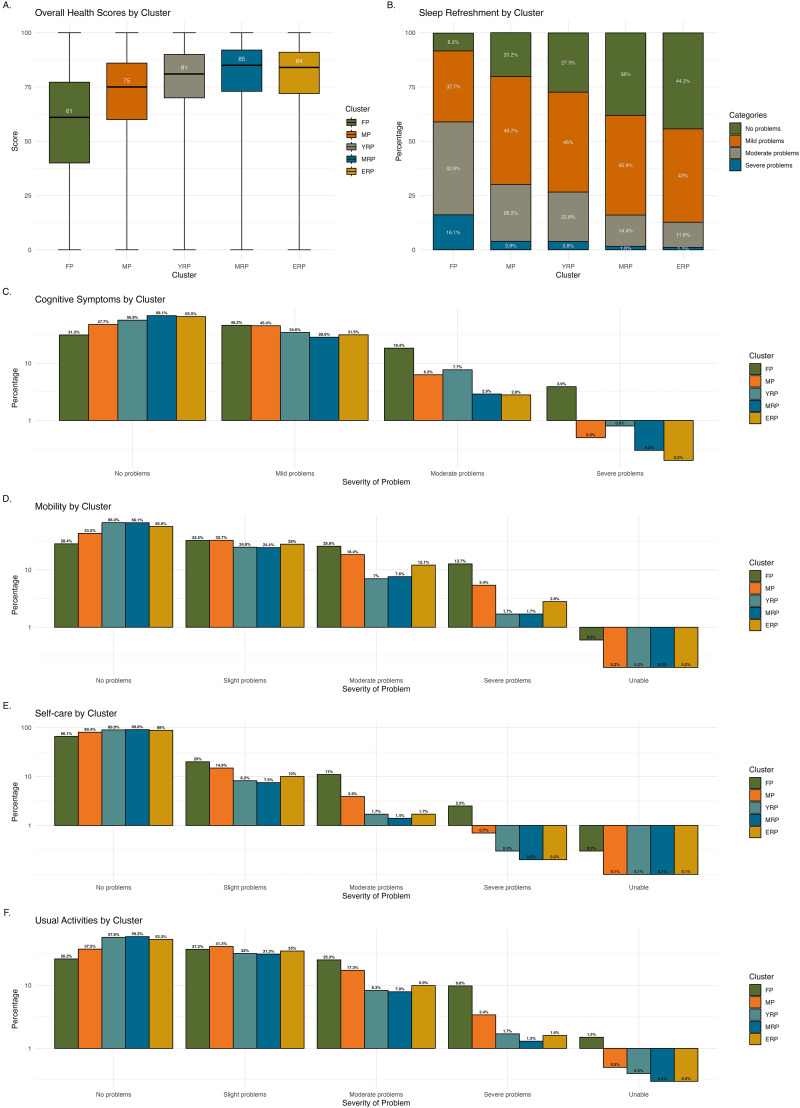
Characteristics of sleep, cognitive functions, and overall health across clusters. illustrates the characteristics of sleep, cognitive functions, and overall health across clusters, including overall health score (Panel A), sleep refreshment (Panel B), cognitive symptoms (Panel C), mobility (Panel D), self-care (Panel E), and usual activities (Panel F). The *y*-axis in Panels D, E and F are scaled proportionally where values below 1% are represented in expanded format to enhance visibility of smaller percentages.

Figure 5 illustrates the differences in sleep, cognitive functions, mobility, and overall health across clusters. Over half (58.9%) of those in FP reported that waking unrefreshed was a moderate to severe problem, while the majority (over 70%) in other clusters experienced only mild or no issues. More than half in FP and MP reported cognitive symptoms, such as memory, thinking skills, and concentration problems, while about one-third in the remaining clusters reported having cognitive issues. Over 20% in FP described these issues as moderate to severe, a rate that dropped to less than 10% in other clusters.

Approximately one-third of individuals in all clusters reported only mild problems with mobility and usual activities. However, more than 10% in FP experienced severe problems or were unable to perform usual activities. Self-care problems, such as washing and dressing, were reported by about one-third in FP, in contrast to only 10%–20% experienced these issues in other clusters. Overall health was rated the lowest in FP (Median score: 61, IQR: 40–77.2), followed by MP (Median score: 75, IQR: 60–86). The median health scores in the other clusters were above 80.

### Associations between clusters and health-related outcomes

Significant relationships were observed between all health-related outcomes and chronic pain clusters, with FP serving as the reference group (Additional file 3). Across depression, sleep, cognition, fatigue, mobility, and overall health, memberships in MP, YRP, MRP and ERP were significantly associated with a decreased relative risk of adverse outcomes compared to FP. Particularly, memberships in MRP and ERP were associated with the lowest likelihood of experiencing adverse outcomes, suggesting protective benefits. Membership in YRP, characterised by younger age groups, appeared to have the lowest relative risk of mobility problems.

### Future directions for persona development

The clustering analysis identified five clusters with varying characteristics and health-related outcomes. Specifically, FP and MP exhibited higher prevalence of adverse health-related outcomes, whereas YRP, MRP, and ERP demonstrated less severity and protective benefits in their memberships. These clusters provided initial insights for data personas detailed in Table 2. The next step in persona development, guided by the personas lifecycle,^9^ is to involve key stakeholders in consultations to create, refine and validate these chronic pain personas. The overarching considerations for developing chronic pain personas for targeted DHIs include:

1. **Clarify the purpose of personas**: Clearly define why personas are needed and how they will be used to guide the design, development, implementation, and evaluation of targeted DHIs.
2. **Data sources and availability**: Determine what data are needed and what type of data are available, whether from previous studies or newly collected information relevant to the context. Outline how these data sources will inform the personas and identify any gaps that need to be addressed.
3. **Stakeholder involvement**: Engage with a diverse group of stakeholders to identify who will be involved in developing chronic pain personas. Determine that their roles in persona development are reflective of real-world experiences and contribute effectively to the specific needs of each subgroup.
4. **Persona creation, refinement, and validation**: Co-design the personas with stakeholders. Outline personas with specific data points, adding sub-personas as needed. Prioritise essential information, determine the appropriate number of personas, and enrich them with individualised narratives to reflect the target population and are practical for use. Validate these personas with stakeholders to ensure that they are representative and adaptable across various stages of the DHI lifecycle.
5. **Communication, implementation, evaluation plan**: Develop practical strategies on how personas will be disseminated and integrated throughout the DHI lifecycle. This also includes establishing plans for ongoing updates and evaluations involving the project team and stakeholders, as well as defining methods to assess the effectiveness of the personas.

**Table 2.** Preliminary insights of data personas.

Clusters	Preliminary data personas
Fibromyalgia-like pain	Female over 60 years old Have pain all over the body for more than five years Live with more than three pain-associated conditions, most likely osteoarthritis Poorer health quality of life, characterised by greater depression, fatigue, and difficulties with daily activities and social relationships From diverse ethnic backgrounds and socioeconomically more disadvantaged
Multisite pain	Female over 70 years old Have pain at multiple sites Have lived with pain for more than five years Live with at least one pain-associated condition, commonly osteoarthritis Experience mild to moderate issues with health-related outcomes, such as fatigue and impacts on sleep and mobility
Younger with regional pain	Female in her 50s Have pain in two or more locations Have lived with pain for less than five years Live with one or two pain-associated conditions most commonly osteoarthritis and migraine Experience mild issues with sleep, fatigue, and depression From diverse ethnic backgrounds and socioeconomically more disadvantaged
Middle age with regional pain	Female in her 60s Have pain in one or two locations Have lived with pain for less than five years With one or two pain-associated conditions, likely osteoarthritis but also rheumatoid arthritis Better health quality of life, but still experiences mild issues with daily activities, sleep, and fatigue
Elderly with regional pain	Male in his 70s Have pain in one or two locations Have lived with pain for less than five years Live with one or two pain-associated conditions, likely osteoarthritis but also gout and rheumatoid arthritis Experience mild issues with sleep, mobility, daily activities, and cognitive skills From more socioeconomically advantaged backgrounds

## Discussion

This study used the UK Biobank to identify distinct chronic pain clusters with the aim of informing persona development for DHIs that provide support targeted at subgroup levels. Our findings demonstrated the use of clustering analysis in distinguishing chronic pain subgroups and quantified their differences with multinomial logistic regression. This analysis has led to the creation of preliminary data personas that capture key characteristics of each subgroup. These personas offer insights into the similarities and varied needs within the chronic pain population and serve as foundational tools for developing targeted interventions. We also provided suggestions for the next steps in persona development, including refining personas through stakeholder engagement, integrating qualitative insights, validating personas against real-world patient experiences, and developing practical strategies for how personas will be disseminated and integrated throughout the DHI lifecycle.

### Primary findings

Using the largest UK population-based dataset, we identified five distinct clusters based on age, sex, and number of pain sites. These three variables yielded informative clusters that provide adequate distinctions in terms of demographics, pain characteristics, comorbidities, and impacts on daily functioning. Previous findings have shown that individuals in mid-age and older age groups report pain of longer duration, at more locations, and with more comorbidities.^38^ Females are more prevalent in reporting chronic pain and depression compared to men.^39^ The number of pain sites is also associated with an increased risk of symptom severity, depression, and prolonged pain duration.^40^ Our findings have validated these variables as a robust starting point for defining subgroups. Specifically, individuals categorised by widespread pain, experiencing pain at multiple locations, report longer durations of pain compared to those in the regional pain category, who have pain at fewer sites. Females are predominantly found in clusters characterised by widespread pain.

The identified clusters share characteristics that are impacted by chronic pain but also display distinct health-related outcomes, indicating the need for differentiated treatment approaches and modalities. Particularly, sleep, mobility, and daily activities are consistently reported as impacted at mild to moderate levels across all clusters. Conversely, fatigue and depression show distinct impacts on daily functioning, with their severity more pronounced in individuals within clusters characterised by widespread pain. Having fewer pain sites appears to act as a protective factor against the severity of pain impacts across all clusters. Multinomial logistic regression further supports that those experiencing pain at fewer locations have the lowest relative risk of adverse health-related outcomes. Our findings are consistent with a previous study investigating pain site clusters in a middle-aged osteoarthritis cohort, where a higher number of pain sites was significantly associated with anxiety, depression, cognitive complaints, and sleep problems.^23^ Females were more likely than males to have a higher number of pain sites. However, in contrast to the previous study, which found younger participants (aged 50–64 years) were more likely to experience more pain sites, while older participants (65 + years) experienced fewer pain sites, our study found that younger participants (under 60 years) experience fewer pain sites. This may reflect differences in cohort characteristics. Future studies should explore subclusters to better target distinct pain profiles.

Our clustering analysis has generated preliminary data personas representing distinct subgroups within the chronic pain population in the UK Biobank. These findings serve as a foundational step in the persona development process, providing a populational data-driven basis for understanding variations in pain profiles. The next steps are involving key stakeholders to create, refine, and validate these chronic pain personas. Specifically, we recommend further exploration in aspects such as age, pain sites, and the impacts of depression, fatigue, and interference on daily functioning when conducting user research to capture the specific needs and challenges faced by each subgroup. Targeted treatment using a risk-stratified approach has been shown to be more effective than standardised care in managing low back pain.^41^ DHIs increasingly aim to deliver personalised interventions tailored at the individual level, though their efficacy and effectiveness have not consistently been proven to be superior to standardised interventions.^42^ Future DHIs for chronic pain may benefit from our findings in enhancing the design and customisation of interventions.

### Strengths and limitations

The primary strength of our study lies in using large population-based data from the UK Biobank to identify distinct and informative clusters, serving as the first step towards persona development for targeted DHIs. This is further complemented by multinomial logistic regression, which quantifies differences between chronic pain clusters and health-related outcomes. However, several limitations need to be addressed. Firstly, the UK Biobank collects primarily epidemiological data, which lacks specific details crucial for intervention development, such as motivations, beliefs, and goals. Additionally, the chronic pain survey does not fully capture the range of pain impacts, with some questions being repetitive or conflated, thereby limiting the dimensions of pain impact we could analyse. Lastly, the predominance of white ethnicities and middle-aged groups in the study population constrains the generalisability of our findings, potentially overlooking cultural and sociodemographic differences that could influence pain experiences.

## Conclusion

The five chronic pain clusters identified from the UK biobank demonstrate informative distinctions. Our analysis reveals that age, sex, and the number of pain sites are critical variables for distinguishing between subgroups. All clusters share similar challenges in sleep, mobility, and daily activities, whereas fatigue and depression show distinct differences, with their impacts more pronounced in widespread pain clusters than in regional pain clusters. Further development of chronic pain persona for targeted DHIs may benefit from this high-level analysis. The next steps should focus on involving key stakeholders to refine and validate these preliminary data personas.

## Supplemental Material

sj-docx-1-dhj-10.1177_20552076251333497 - Supplemental material for Identifying chronic pain subgroups in the UK biobank for persona development: A clustering analysis Supplemental material, sj-docx-1-dhj-10.1177_20552076251333497 for Identifying chronic pain subgroups in the UK biobank for persona development: A clustering analysis by Ting-Chen Chloe Hsu, Pauline Whelan, Christopher J Armitage and John McBeth in DIGITAL HEALTH
