# Supplementary material for: Identifying chronic pain subgroups in the UK biobank for persona development: A clustering analysis
Source: Digit Health. 2025 May 14;11:20552076251333497. doi: 10.1177/20552076251333497 (PMC12078971; doi:10.1177/20552076251333497)
Supplement: sj-docx-1-dhj-10.1177_20552076251333497 - Supplemental material for Identifying chronic pain subgroups in the UK biobank for persona development: A clustering analysis [file sj-docx-1-dhj-10.1177_20552076251333497.docx]

**Additional file 1 Dissimilarity cost of different cluster (k) solutions**
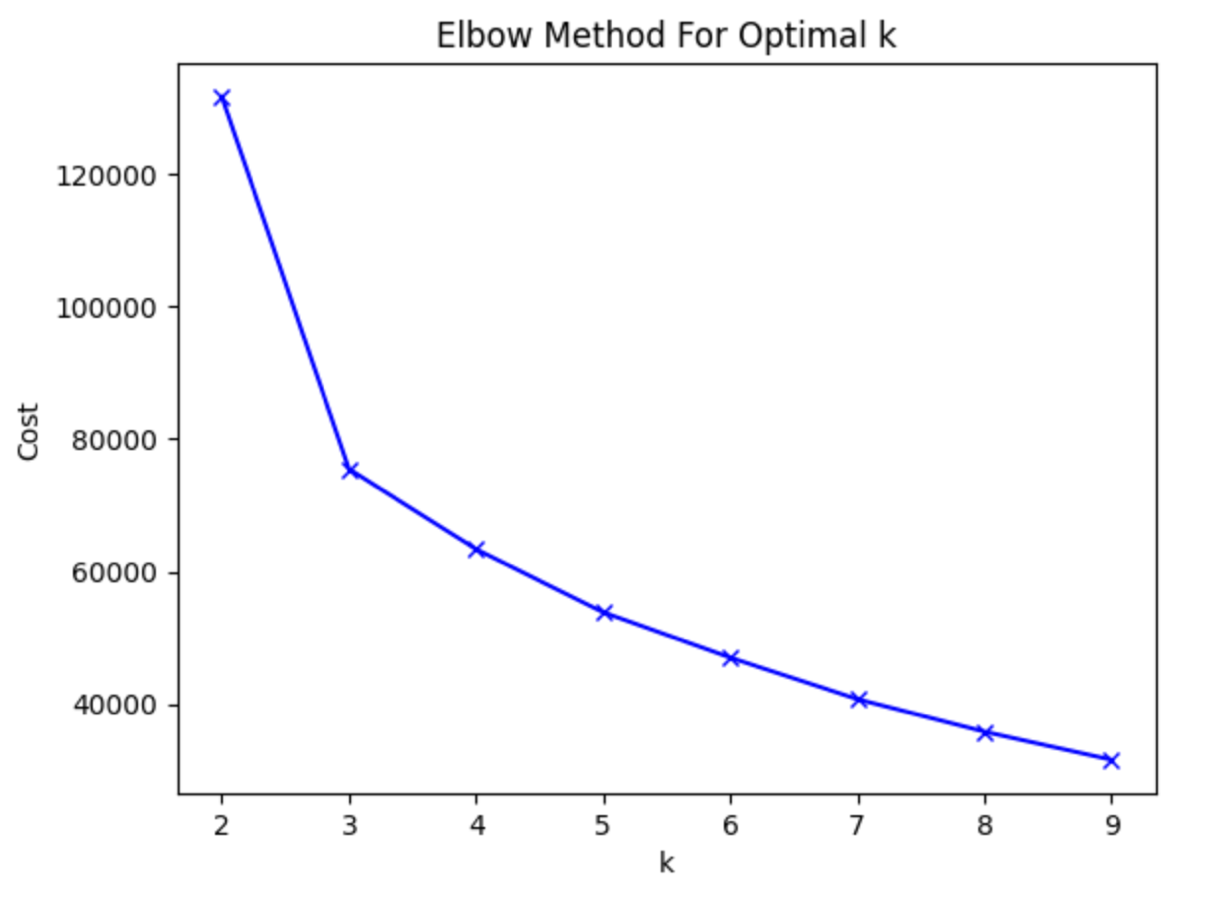


Figure legend: this figure shows the dissimilarity cost for each cluster k, where an elbow point occurs at cluster five. Further increases in the number of clusters result in diminishing improvements.

**Additional file 2** **Cluster profiles based on demographics, pain information, and health outcomes**

|  | **FP** | **MP** | **YRP** | **MRP** | **ERP** |
| --- | --- | --- | --- | --- | --- |
| Number of participants, n (%) | 10,045 (11.2%) | 16,047 (17.9%) | 19,721 (21.9%) | 22,907 (25.5%) | 21,133 (23.5%) |
| **Demographics** | | | | | |
| Female, n (%) | 7,202 (71.7) | 12,435 (77.5) | 12,308 (62.4) | 15,339 (67) | 6,955 (32.9) |
| Median age (IQR) | 66.3 (60.1-72.1) | 71.8 (69.3-74.7) | 56.1 (53.6-58.5) | 65 (62.9-67.1) | 73.6 (71.5-76.2) |
| Ethnicity, n (%) |  |  |  |  |  |
| White | 9,479 (94.4) | 15,699 (97.8) | 18,703 (94.8) | 22,269 (97.2) | 20,766 (98.3) |
| Mixed | 75 (0.7) | 53 (0.3) | 202 (1) | 103 (0.4) | 39 (0.2) |
| Asian or Asian British | 150 (1.5) | 86 (0.5) | 249 (1.3) | 159 (0.7) | 106 (0.5) |
| Black or Black British | 172 (1.7) | 57 (0.4) | 281 (1.4) | 144 (0.6) | 32 (0.2) |
| Chinese | 31 (0.3) | 20 (0.1) | 69 (0.3) | 59 (0.3) | 17 (0.1) |
| Other ethnic groups | 96 (1) | 79 (0.5) | 151 (0.8) | 121 (0.5) | 68 (0.3) |
| Age completed full-time education, Median (IQR) | 16 (15-17) | 16 (15-18) | 17 (16-18) | 17 (16-18) | 16 (15-18) |
| **Pain sites** | | | | | |
| Median (IQR) | 14 (14-14) | 6 (5-7) | 3 (2-5) | 3 (2-4) | 2 (2-3) |
| Minimum number | 9 | 4 | 0 | 0 | 0 |
| Maximum number | 14 | 11 | 11 | 7 | 6 |
| **Duration of pain, n (%)** | | | | | |
| 3-12 months | 986 (9.8) | 2,774 (17.3) | 5,332 (27) | 6,352 (27.7) | 6,371 (30.1) |
| 1-5 years | 2,732 (27.2) | 6,085 (37.9) | 7,506 (38.1) | 9,081 (39.6) | 8,297 (39.3) |
| More than 5 years | 6,281 (62.5) | 7,084 (44.1) | 6,765 (34.3) | 7,358 (32.1) | 6,344 (30) |
| **Number of pain-associated conditions, n (%)** | | | | | |
| 0 | 853 (8.5) | 2,007 (12.5) | 7,364 (37.3) | 7,282 (31.8) | 6,071 (28.7) |
| 1 | 2,174 (21.6) | 4,893 (30.5) | 6,715 (34) | 8,835 (38.6) | 8,138 (38.5) |
| 2 | 2,437 (24.3) | 4,525 (28.2) | 3,567 (18.1) | 4,507 (19.7) | 4,541 (21.5) |
| 3+ | 4,581 (45.6) | 4,622 (28.8) | 2,075 (10.5) | 2,283 (9.9) | 2,383 (11.1) |
| **Depression** | | | | | |
| Had depression in the past 6 months, n (%) | 3,822 (38) | 3,454 (21.5) | 4,068 (20.6) | 2,915 (12.7) | 1,958 (9.3) |
| PHQ9, median (IQR) | 6 (3-11) | 3 (1-6) | 3 (1-6) | 2 (0-4) | 1 (0-3) |
| PHQ9 categories, n (%) | | | | | |
| None (0-4) | 3,895 (38.8) | 1,0179 (63.4) | 12,192 (61.8) | 17,636 (77) | 17,527 (82.9) |
| Mild (5-9) | 3,154 (31.4) | 4,122 (25.7) | 4,970 (25.2) | 3,978 (17.4) | 2,911 (13.8) |
| Moderate (10-14) | 1,618 (16.1) | 1,196 (7.5) | 1,564 (7.9) | 872 (3.8) | 511 (2.4) |
| Moderately Severe (15-19) | 779 (7.8) | 394 (2.5) | 639 (3.2) | 293 (1.3) | 143 (0.7) |
| Severe (20-27) | 599 (6) | 156 (1) | 356 (1.8) | 128 (0.6) | 41 (0.2) |
| Levels of difficulty depression has caused, n (%) | | | | | |
| Not difficult at all | 3,618 (36) | 8,263 (51.5) | 10,100 (51.2) | 12,100 (52.8) | 9,975 (47.2) |
| Somewhat difficult | 4,246 (42.3) | 5,069 (31.6) | 5,532 (28.1) | 4,394 (19.2) | 3,987 (18.9) |
| Very difficult | 890 (8.9) | 463 (2.9) | 540 (2.7) | 301 (1.3) | 254 (1.2) |
| Extremely difficult | 599 (6) | 181 (1.1) | 287 (1.5) | 131 (0.6) | 59 (0.3) |
| **Fatigue** | | | | | |
| Had fatigue for at least 6 months, n (%) | 6,420 (63.9) | 5,450 (34) | 6,370 (32.3) | 4,602 (20.1) | 3,693 (17.5) |
| Fatigue interferes with work, family or social life, n (%) | | | | | |
| Disagree (1-3) | 3,263 (32.5) | 8,253 (51.4) | 8,141 (41.3) | 9,539 (41.6) | 8,878 (42) |
| Neutral (4) | 1,100 (11) | 1,462 (9.1) | 1,308 (6.6) | 1,132 (4.9) | 1,127 (5.3) |
| Agree (5-7) | 4,836 (48.1) | 3,053 (19) | 4,172 (21.2) | 2,586 (11.3) | 1,915 (9.1) |
| Fatigue interferes with physical functioning, n (%) | | | | | |
| Disagree (1-3) | 2,159 (21.5) | 5,725 (35.7) | 5,746 (29.1) | 6,913 (30.2) | 6,630 (31.4) |
| Neutral (4) | 998 (9.9) | 1,843 (11.5) | 1,714 (8.7) | 1,552 (6.8) | 1,538 (7.3) |
| Agree (5-7) | 6,053 (60.3) | 5,204 (32.4) | 6,166 (31.3) | 4,789 (20.9) | 3,750 (17.7) |
| Fatigue is among three most disabling symptoms, n (%) | | | | | |
| Disagree (1-3) | 3,366 (33.5) | 8,331 (51.9) | 8,774 (44.5) | 9,535 (41.6) | 8,544 (40.4) |
| Neutral (4) | 1,041 (10.4) | 1,316 (8.2) | 1,202 (6.1) | 1,028 (4.5) | 1,093 (5.2) |
| Agree (5-7) | 4,782 (47.6) | 3,106 (19.4) | 3,623 (18.4) | 2,678 (11.7) | 2,245 (10.6) |
| **Sleep** | | | | | |
| Waking unrefreshed, n (%) | | | | | |
| No problems | 828 (8.2) | 3,235 (20.2) | 5,391 (27.3) | 8,696 (38) | 9,343 (44.2) |
| Mild problems | 3,288 (32.7) | 7,971 (49.7) | 9,076 (46) | 10,525 (45.9) | 9,087 (43) |
| Moderate problems | 4,304 (42.8) | 4,197 (26.2) | 4,488 (22.8) | 3,296 (14.4) | 2,444 (11.6) |
| Severe problems | 1,613 (16.1) | 627 (3.9) | 755 (3.8) | 377 (1.6) | 243 (1.1) |
| **Cognitive symptoms** | | | | | |
| Memory, thinking skills and/or concentration, n (%) | | | | | |
| No problems | 3,145 (31.3) | 7,653 (47.7) | 11,198 (56.8) | 15,605 (68.1) | 13,834 (65.5) |
| Mild problems | 4,637 (46.2) | 7,284 (45.4) | 6,829 (34.6) | 6,550 (28.6) | 6,648 (31.5) |
| Moderate problems | 1,853 (18.4) | 1,012 (6.3) | 1,520 (7.7) | 666 (2.9) | 602 (2.8) |
| Severe problems | 393 (3.9) | 82 (0.5) | 161 (0.8) | 70 (0.3) | 34 (0.2) |
| **Health quality of life (EQ-5D-5L)** | | | | | |
| Overall health (0-100), median (IQR) | 61 (40-77.2) | 75 (60-86) | 81 (70-90) | 85 (73-92) | 84 (72-91) |
| Problems with mobility, n (%) | | | | | |
| No problems | 2,853 (28.4) | 6,940 (43.2) | 13,086 (66.4) | 15,138 (66.1) | 12,029 (56.9) |
| Slight problems | 3,263 (32.5) | 5,249 (32.7) | 4,898 (24.8) | 5,589 (24.4) | 5,915 (28) |
| Moderate problems | 2,596 (25.8) | 2,958 (18.4) | 1,378 (7) | 1,742 (7.6) | 2,561 (12.1) |
| Severe problems | 1,272 (12.7) | 865 (5.4) | 327 (1.7) | 398 (1.7) | 594 (2.8) |
| Unable | 61 (0.6) | 35 (0.2) | 32 (0.2) | 40 (0.2) | 34 (0.2) |
| Problems with self-care, n (%) | | | | | |
| No problems | 6,641 (66.1) | 12,900 (80.4) | 17,700 (89.8) | 20,809 (90.8) | 18,594 (88) |
| Slight problems | 2,013 (20) | 2,392 (14.9) | 1,621 (8.2) | 1,728 (7.5) | 2,121 (10) |
| Moderate problems | 1,101 (11) | 633 (3.9) | 328 (1.7) | 320 (1.4) | 365 (1.7) |
| Severe problems | 255 (2.5) | 106 (0.7) | 62 (0.3) | 37 (0.2) | 39 (0.2) |
| Unable | 35 (0.3) | 16 (0.1) | 10 (0.1) | 13 (0.1) | 14 (0.1) |
| Problems with usual activities, n (%) | | | | | |
| No problems | 2,634 (26.2) | 6,016 (37.5) | 11,363 (57.6) | 13,568 (59.2) | 11,256 (53.3) |
| Slight problems | 3,734 (37.2) | 6,627 (41.3) | 6,313 (32) | 7,160 (31.3) | 7,391 (35) |
| Moderate problems | 2,545 (25.3) | 2,776 (17.3) | 1,627 (8.3) | 1,818 (7.9) | 2,083 (9.9) |
| Severe problems | 981 (9.8) | 547 (3.4) | 332 (1.7) | 297 (1.3) | 337 (1.6) |
| Unable | 151 (1.5) | 81 (0.5) | 86 (0.4) | 64 (0.3) | 66 (0.3) |

**Additional file 3 Associations of chronic pain clusters with health outcomes**

|  | **FP**  **(n = 10,045)** | **MP**  **(n = 16,047)** | **YRP**  **(n = 19,721)** | **MRP**  **(n = 22,907)** | **ERP**  **(n = 21,133)** |
| --- | --- | --- | --- | --- | --- |
|  | **RRR (95% CI)** | | | | |
| **Depression** | | | | | |
| Had depression in the past 6 months | 1.00 | 0.44 (0.42-0.47) | 0.42 (0.4-0.44) | 0.23 (0.22-0.25) | 0.16 (0.15-0.17) |
| PHQ-9 categories | | | | | |
| None (reference) | 1.00 |  |  |  |  |
| Mild | 1.00 | 0.5 (0.47-0.53) | 0.5 (0.48-0.53) | 0.28 (0.26-0.3) | 0.21 (0.19-0.22) |
| Moderate | 1.00 | 0.28 (0.26-0.31) | 0.31 (0.29-0.33) | 0.12 (0.11-0.13) | 0.07 (0.06-0.08) |
| Moderately Severe | 1.00 | 0.19 (0.17-0.22) | 0.26 (0.24-0.29) | 0.08 (0.07-0.1) | 0.04 (0.03-0.05) |
| Severe | 1.00 | 0.1 (0.08-0.12) | 0.19 (0.17-0.22) | 0.05 (0.04-0.06) | 0.02 (0.01-0.02) |
| Difficulty of depression | | | | | |
| Not at all (reference) | 1.00 |  |  |  |  |
| Somewhat difficult | 1.00 | 0.52 (0.49-0.55) | 0.47 (0.44-0.49) | 0.31 (0.29-0.33) | 0.34 (0.32-0.36) |
| Very difficult | 1.00 | 0.23 (0.20-0.26) | 0.22 (0.19-0.24) | 0.10 (0.09-0.12) | 0.10 (0.1-0.12) |
| Extremely difficult | 1.00 | 0.13 (0.11-0.16) | 0.17 (0.15-0.2) | 0.07 (0.05-0.08) | 0.04 (0.03-0.05) |
| **Sleep** | | | | | |
| Waking unrefreshed | | | | | |
| No (reference) | 1.00 |  |  |  |  |
| Mild problem | 1.00 | 0.62 (0.57-0.68) | 0.42 (0.39-0.46) | 0.31 (0.28-0.33) | 0.25 (0.23-0.27) |
| Moderate problem | 1.00 | 0.25 (0.23-0.27) | 0.16 (0.15-0.17) | 0.07 (0.07-0.08) | 0.05 (0.05-0.06) |
| Severe problem | 1.00 | 0.1 (0.09-0.11) | 0.07 (0.06-0.08) | 0.02 (0.02-0.03) | 0.01 (0.01-0.02) |
| **Cognition** | | | | | |
| Cognitive symptoms | | | | | |
| No (reference) | 1.00 |  |  |  |  |
| Mild problem | 1.00 | 0.65 (0.61-0.68) | 0.41 (0.39-0.44) | 0.29 (0.27-0.3) | 0.33 (0.31-0.34) |
| Moderate problem | 1.00 | 0.22 (0.21-0.25) | 0.23 (0.21-0.25) | 0.07 (0.07-0.08) | 0.07 (0.07-0.08) |
| Severe problem | 1.00 | 0.09 (0.07-0.11) | 0.12 (0.1-0.12) | 0.04 (0.03-0.05) | 0.02 (0.01-0.03) |
| **Fatigue** | | | | | |
| Fatigue lasted for 6 months | 1.00 | 0.28 (0.26-0.29) | 0.26 (0.25-0.27) | 0.13 (0.13-0.14) | 0.11 (0.11-0.12) |
| Fatigue interferes with physical functioning | 1.00 | 0.74 (0.73-0.75) | 0.76 (0.75-0.77) | 0.68 (0.67-0.69) | 0.65 (0.64-0.66) |
| Fatigue is among three most disabling symptoms | 1.00 | 0.74 (0.73-0.75) | 0.74 (0.73-0.75) | 0.67 (0.66-0.68) | 0.67 (0.66-0.68) |
| Fatigue interferes with work, family or social life | 1.00 | 0.72 (0.71-0.73) | 0.76 (0.75-0.77) | 0.65 (0.64-0.66) | 0.63 (0.62-0.63) |
| **Health outcomes** | | | | | |
| Mobility | | | | | |
| No (reference) | 1.00 |  |  |  |  |
| Slight problem | 1.00 | 0.66 (0.62-0.70) | 0.33 (0.31-0.35) | 0.32 (0.3-0.34) | 0.43 (0.41-0.46) |
| Moderate problem | 1.00 | 0.47 (0.44-0.5) | 0.12 (0.11-0.13) | 0.13 (0.12-0.14) | 0.23 (0.22-0.25) |
| Severe problem | 1.00 | 0.28 (0.25-0.31) | 0.06 (0.05-0.06) | 0.06 (0.05-0.07) | 0.11 (0.1-0.12) |
| Unable | 1.00 | 0.24 (0.16-0.36) | 0.11 (0.07-0.18) | 0.12 (0.08-0.18) | 0.13 (0.09-0.2) |
| Self-care | | | | | |
| No (reference) | 1.00 |  |  |  |  |
| Slight problem | 1.00 | 0.61 (0.57-0.65) | 0.3 (0.28-0.32) | 0.27 (0.26-0.29) | 0.38 (0.35-0.4) |
| Moderate problem | 1.00 | 0.3 (0.27-0.33) | 0.11 (0.1-0.13) | 0.09 (0.08-0.11) | 0.12 (0.11-0.14) |
| Severe problem | 1.00 | 0.21 (0.17-0.27) | 0.09 (0.07-0.12) | 0.05 (0.03-0.07) | 0.06 (0.04-0.08) |
| Unable | 1.00 | 0.24 (0.13-0.43) | 0.11 (0.05-0.22) | 0.12 (0.06-0.22) | 0.14 (0.08-0.27) |
| Usual activities | | | | | |
| No (reference) | 1.00 |  |  |  |  |
| Slight problem | 1.00 | 0.78 (0.73-0.83) | 0.39 (0.37-0.42) | 0.37 (0.35-0.39) | 0.46 (0.44-0.49) |
| Moderate problem | 1.00 | 0.48 (0.45-0.51) | 0.15 (0.14-0.16) | 0.14 (0.13-0.15) | 0.19 (0.18-0.21) |
| Severe problem | 1.00 | 0.24 (0.22-0.27) | 0.08 (0.07-0.09) | 0.06 (0.05-0.07) | 0.08 (0.07-0.09) |
| Unable | 1.00 | 0.24 (0.18-0.31) | 0.13 (0.1-0.17) | 0.08 (0.06-0.11) | 0.1 (0.08-0.14) |
| Overall health | 1.00 | 1.02 (1.02-1.02) | 1.04 (1.04-1.04) | 1.05 (1.05-1.05) | 1.05 (1.05-1.05) |
